# Development and testing of innovative patient resources for the management of coronary heart disease (CHD): a descriptive study

**DOI:** 10.1186/1472-6963-6-95

**Published:** 2006-08-06

**Authors:** Julie Redfern, Elizabeth Ellis, Tom Briffa, Saul B Freedman

**Affiliations:** 1 School of Physiotherapy, University of Sydney, Sydney, Australia; 2 School of Physiotherapy, Curtin University of Technology, Perth, Australia; 3 Department of Cardiology, Concord Hospital, Sydney, Australia

## Abstract

**Background:**

Although heart disease is a major cause of morbidity and mortality the majority of patients do not access existing rehabilitation programs and patient resources are not designed to facilitate patient choice and decision-making. The objective of this study was to develop and test a series of risk factor modules and corresponding patient information leaflets for secondary prevention of CHD.

**Methods:**

In phase one, a series of risk factor modules and management options were developed following analysis of literature and interviews with health professionals. In phase two, module information leaflets were developed using published guidelines and interviews of people with CHD. In phase three, the leaflets were tested for quality (DISCERN), readability (Flesch) and suitability (SAM) and were compared to the existing cardiac rehabilitation (CR) information leaflet. Finally, the patients assessed the leaflets for content and relevance.

**Results:**

Four key risk factors identified were cholesterol, blood pressure, smoking and physical inactivity. Choice management options were selected for each risk factor and included medical consultation, intensive health professional led program, home program and self direction. Patient information needs were then identified and leaflets were developed. DISCERN quality scores were high for cholesterol (62/80), blood pressure (59/80), smoking (62/80) and physical activity (62/80), all scoring 4/5 for overall rating. The mean Flesch readability score was 75, representing "fairly easy to read", all leaflets scored in the superior category for suitability and were reported to be easy to understand, useful and motivating by persons with CHD risk factors. The developed leaflets scored higher on each assessment than the existing CR leaflets.

**Conclusion:**

Using a progressive three phase approach, a series of risk factor modules and information leaflets were successfully developed and tested. The leaflets will contribute to shared-decision making and empowerment for persons with CHD.

## Background

Coronary heart disease (CHD) is a chronic illness that is best managed when positive health behaviors become integrated into long-term lifestyle habits [1]. Secondary prevention of CHD involves risk factor reduction through control of health behaviors such as diet, physical activity, smoking and medication adherence using a coordinated approach and referrals to health professionals [2,3]. Modification of risk factors such as blood cholesterol, blood pressure (BP), physical inactivity, smoking, overweight, diabetes and depression can reduce cardiovascular events, the need for coronary revascularisation and improve quality of life (QOL) [4]. However, strategies are needed to motivate patients to consistently follow multidimensional treatment regimens [1].

Although attendance at cardiac rehabilitation (CR) programs is widely recommended [5,6] there is significant under-use of facility-based programs in Australia [7,8] and internationally [9-11]. Despite clear short-term benefits for attendees, large groups of patients are not accessing, which presents an opportunity and challenge to improve CHD care [10]. Although many authors have suggested the need for development of alternative models, these have not yet been widely described or implemented. One pilot study investigated secondary prevention of CHD using a 'modular' approach [12]. For the study, a metropolitan cardiac rehabilitation program was offered as individual components, or modules, based on patient need rather than an all-or-nothing exercise-based approach. Although only 26 subjects participated, the study demonstrated enhanced completion rates and cost effectiveness. The pilot study was an important step from standard cardiac rehabilitation however, modules were still conducted in a group environment and were based on education without formal goal setting and follow-up and no patient information leaflets were provided. Results of the pilot study suggest that development and evaluation of more detailed unique risk factor modules may enhance long-term outcomes.

The development of patient resources based on multidimensional behavior modification techniques may enhance compliance with long-term risk factor interventions [13]. Techniques include goal setting, choice and offering a wide range of individually-tailored services [6]. For chronic disease, change needs to be conceptualised in a positive light to reduce anxiety rather than focusing on negative aspects of making a change [1]. Setting of mutually-agreed and realistic goals with clear time-frames enhances active patient orientation and motivation which facilitates behavior change [1,14]. The inclusion of a confidence rating for goal achievement may improve the health professional's insight into a patient's motivation to change [1].

Offering choice, in situations where there are several management options, based on individual need and preference is a further method of enhancing active patient orientation [15]. It is also suggested that options offered as a menu of strategies are more successful because it encourages each patient's task to be one of "choosing rather than refuting" [1]. Providing choice allows different people to respond to health professionals in different ways based on each persons needs at different times. We have chosen the term 'guided self-choice' to describe this model which creates a collaborative encounter, which is similar to the spirit of behavior counseling [16].

Facilitation of collaborative relationships, shared decision-making and communication of expectations in a supportive environment helps motivate behavior change and contribute to the disease management process [17,18]. Formation of collaborative relationships decreases patient anxiety and dissatisfaction that is often related to uncertainty and lack of information, explanation and feedback [19]. Studies have demonstrated that encouraging patients to participate in treatment decisions improves health status, QOL [20,21], satisfaction, compliance and treatment outcomes [22]. More specifically, patients with a higher degree of active orientation are more likely to comply with treatment recommendations for BP [18], diabetes and physical activity [23].

Although provision of effective education is an essential component of healthcare [24], patients tend to forget approximately half the information provided [25]. Providing supplementary written information increases patients' knowledge of disease [26-28] and reduces distress [29] and decisional conflict [30] because it reinforces verbal instructions and serves as a home resource [31]. Effective patient information must be evidence-based, clearly presented and most importantly, involve patients throughout the process of development [32-34]. Information materials should succinctly deal with the relevant messages [31] be written in active voice with personalised messages and use simple terminology [14,31,35]. Layout should be clear, simple and consistent [14,35] and use subheadings to allow patients to efficiently sift through the information [17,31].

Various studies have tested patient education materials including those for drug information [36,37], prostate cancer [38], asthma [39], cardiac catheterization [40] and about general practice [41]. Two studies have highlighted the importance of devoting considerable effort to the development of educational materials and including patients throughout the entire process [42,43]. Both studies used a progressive approach and demonstrated that if a leaflet is written at an easily readable level and in collaboration with patients, knowledge is increased. The objectives of this study were to develop and evaluate a series of cardiac risk factor modules and corresponding information leaflets for secondary prevention of CHD.

## Methods

### Phase 1 – Development of secondary prevention risk factor modules

The objective of phase one was to develop a series of CHD secondary prevention risk factor modules each containing a menu of management options. Major CHD risk factors were identified after a systematic search of electronic databases and cross-reference of publications for consistent risk factors that put the greatest proportion of patients at the highest risk. In addition, six health professionals including allied health, medical and nursing, were interviewed about risk factors and their prevention. They were specifically asked which risk factors were the most prevalent, which presented the greatest CHD risk.

Once key risk factors were identified, the authors conducted an investigation of the relevant and available management options within the area health service. Key areas included the availability of lipid clinics, smoking cessation programs and local exercise and walking groups. Searches were conducted via internet searches, telephone contacts with health professionals and in person. Contact details, general information and costs, if applicable, for each service were recorded.

### Phase 2 – Development of module information leaflets

The objective of phase two was to develop information leaflets for each of the modules, developed in phase 1, using published guidelines [10]. Semi-structured interviews of volunteers with at least one CHD risk factor were also conducted to identify their feedback for the leaflets along with ideas to enhance motivation for behavior change. Potential volunteers were identified consecutively from an outpatient clinic at a local hospital, were between 30–60 years and had least one of the relevant CHD risk factors.

Each interview was guided with a series of seven pre-developed questions which focused on the type and volume of information people would like about CHD risk factors and what information would be motivating. All participants were also asked if they wanted to be involved in the management decision-making and the interview was concluded with an open question asking for any further comments.

After systematically addressing each of the published guidelines [17] and using themes generated from interviews of health professionals and people with CHD risk factors, the module information leaflets were drafted. Apart from a heading and relevant logos, additional space was provided for documenting the risk factor level, goal, time frame for achievement and information about the nationally recommended level. The front page also included a series of options, each with a 'tick box', from which the patient could choose a single preferred management option. Each information leaflet also included general information about CHD, suggestions for reducing the risk factor and space for documenting phone numbers and appointment details. A point-form layout with size 12 font in an A5 size book format was chosen for ease of reading and visual appeal.

### Phase 3 – Testing of module information leaflets

The objective of phase three was to evaluate the module information leaflets for quality, readability and suitability and content and relevance. The newly developed leaflets were also compared to the existing CR information leaflet that was obtained from the rehabilitation coordinator. Two independent researchers completed all assessment tools for each leaflet and the mean scores were calculated for each subsection.

Quality of the leaflets was evaluated using the DISCERN instrument [44] which is a 16 item questionnaire and is a reliable and valid measure [44-46] with three sections – reliability of the publication, quality of the information about treatment choices and overall rating. Each item is scored on a 5-point Likert scale from one (low quality) to five (high quality). An aggregate score greater than 75 represents very high quality and a final score of less than 15 represents very low quality [44].

Readability of the leaflets was evaluated using the Flesch readability formula which assesses the average number of syllables per word and the average number of words per sentence [47]. The final score for reading ease are out of a possible 100, scores between 70 and 100 represent matter that is understandable by people who have completed the fourth grade of schooling [47]. The human interest score represents the amount of personalisation in the piece of writing, scores range from zero (no human interest) to 100 (extensive personal interest). The Flesch formula has been demonstrated to be reliable and highly correlated with other readability formulae for analysis of health-based literature [48].

The leaflets were evaluated for suitability using the Suitability Assessment of Materials (SAM) instrument [49]. The SAM has 22-items that test six criteria of written materials – content, literacy demand, graphics, presentation, learning stimulation or motivation and cultural appropriateness. Final aggregate sores greater than 70% are judged to be superior, scores between 40%–69% are deemed adequate and scores below 40% are considered unsuitable. The SAM has been validated following evaluation of a nutritional leaflet by more than 150 health professionals [49].

To evaluate content, relevance and usefulness of the leaflets from a patient's perspective, a group of people with at least one CHD risk factor completed a questionnaire. The questionnaire was completed anonymously in the hospital outpatient clinic and was made up of nine Likert items across four sections – overall rating, heart disease information, treatment options and style and readability. The questionnaire was designed to obtain opinions on core attributes previously outlined [17] – content adequacy (understanding), relevance (a balanced view) and matching of information with the individual's experience (visual appeal). Readers were asked to rate each question on a five-point visual analogue scale, higher scores represented more positive responses. Space was also provided for comments and identification of words not understood.

A target recruitment rate of 10 participants per risk factor was set, providing a total of 40 participants across the four risk factors. Participants were recruited consecutively from a local hospital clinic and were included if they had at least one of the relevant CHD risk factor and if they volunteered to review the leaflet. The results for the newly developed and the existing CR leaflets were compared using t-tests for continuous variables and chi-squared for proportions, two-tailed p-values of < 0.05 were considered significant.

### Ethical approval

Ethical approval for the study was provided by the Central Sydney Area Health Service CRGH Zone and the University of Sydney. Written and informed consent was obtained from all participants prior to commencement.

## Results

### Phase 1 – Development of secondary prevention risk factor modules

Following literature analysis and interviews with health professionals, four key CHD risk factors were chosen for development into modules and corresponding information leaflets. The risk factors chosen were cholesterol management as well as BP-lowering, smoking cessation and physical inactivity which could be chosen based on need and preference. High cholesterol was chosen because it is a very highly prevalent and continuous risk factor where the higher the value, the higher the risk. In Australia, about half the adult population have high cholesterol [50]. Importantly, cholesterol-lowering decreases the relative risk for major coronary events by 27%, for CHD mortality by 27% and for all cause mortality by 21% [51]. For these reasons along with the strong recommendation of the Cardiologist interviewed and based on the secondary prevention benefit of cholesterol-lowering, it was decided that cholesterol lowering would be a priority risk factor module that all patients should be encouraged to participate in.

High BP was chosen because of its prevalence and the graded relationship between BP and risk of CHD risk [52]. Approximately 28% of the Australian adult population have high BP [50]. BP lowering has been found to cause a 12% reduction in cardiovascular events and a 22% reduction in mortality [53]. Smoking was a chosen key risk factor because it accounts for approximately 13% of Australian deaths annually [54]. In addition, smoking cessation has both dose dependent and reversible improvements on endothelial function [55]. Physical activity was also chosen because 44% of the Australian adult population do not participate in a level of physical activity that is sufficient to achieve a health benefit [54]. In addition, increasing physical activity levels can have a positive effect on other risk factors such as cholesterol, BP, overweight, diabetes and depressed mood [56].

Four common management options were identified were – medical consultation, a health professional-led intervention, a home program and self help (Table 2). Where practical, each management option was incorporated into each module to provide a menu-style choice. For the medical consultation, patients were to be encouraged to initiate contact with their doctor, to record and follow their advice. For health professional-led intervention, the risk factor was managed by participation in a supervised, structured program that may be hospital or community based. For the home program option, patients were to manage the risk factor through participation in a home-based program. Finally, in the self-help option, patients could choose to manage the risk factor through their own means and were provided with sources of local information including the internet, telephone numbers and books. At the completion of phase 1, although all patients were to be encouraged to participate in cholesterol-lowering, each module followed a guided self-choice approach where patients could choose, in collaboration with a health professional, the management option to suit their individual needs or preference.

### Phase 2 – Development of module information leaflets

Common themes identified from published guidelines [17] were that the leaflets should be simple and positive in design and language, repeat key terms, clearly identify treatment options and importantly that patients should be involved throughout development and testing. A group of 12 people with at least one CHD risk factor volunteered to be interviewed, nine were male, age ranged from 30–52 years with a mean of 47 years. Seven participants had experienced previous angina, one had a previous myocardial infarction and three had no previous acute coronary syndrome (ACS). Common content-related themes included widespread request to have basic information that provided reassurance as well as realistic and practical methods for managing the risk factor (Table 1). Participants suggested the use of a positive and motivating tone, most felt strongly about the provision of contact details and the need for space to write questions and all commented on the need for simplicity and brevity.

Of the participants with high cholesterol, 100% wanted to know their own and the recommended level and 66% wanted information about cholesterol testing and medications. Of the participants with high BP, 100% requested information about the effects of BP on the heart and about medications for BP. Of the patients who were physically inactive, 100% expressed a need for simple and practical information about safe activities. Of the current smokers interviewed, 100% said they wanted information about how to quit and none wanted information about why. For all risk factors, 100% of participants felt strongly about the need for shared decision-making and tailoring management to suit their lifestyle.

### Phase 3 – Testing of module information leaflets (Table 3 and Table 4)

The final calculated results relating to leaflet quality, out of a possible 80, for the DISCERN were 61.5 for blood cholesterol, 59 for blood pressure, 62 for smoking and 61.5 for physical activity. All leaflets scored 4/5 for overall rating and the mean results for each of the three key DISCERN sections was calculated (Table 3). Flesch readability scores for the four leaflets ranged from 68 to 82 with a mean of 75. The overall score for the combined module leaflets was 75, placing them in the "fairly easy to read" category [47]. When tested for suitability, all four module information leaflets scored greater than an 80% SAM rating placing them all in the superior category. Compared to the existing CR information leaflet, the newly developed module leaflets scored significantly higher on the DISCERN (p = 0.04), Flesch (p = 0.01) and SAM (p < 0.001) assessment tools.

A total of 40 participants, 10 for each module, tested the leaflets for content and relevance. Demographic data and the questionnaire results for each module were summarised, scores were all out of a possible five (Table 4). General suggestions given by participants relating to presentation and understanding included increasing the space between the goal and management options, providing more space to write notes and placing the goal and measured risk factor in a shaded box to enhance clarity. Specific comments – "motivates me to lower my cholesterol", "sufficient information", "easy to understand", "clear language", "options are clear and in a logical order" and "providing a variety of options is more motivating than someone just telling me what to do". One participant believed the term "cessation" on the smoking leaflet should be changed to "quitting". All changes suggested by the volunteers were incorporated into the leaflets.

## Discussion

This study describes the development and testing of a new series of CHD risk factor modules and corresponding information leaflets for patients. From the literature and input from consumers and health professionals, it was determined that cholesterol-lowering would be a mandatory module and remaining risk factors (BP management, physical inactivity and smoking cessation) would be optional modules. A menu of management options was developed and adapted for each of the risk factors to facilitate active choice rather than encourage refuting and scrutinising that often occurs if options are presented one by one [1].

Development of the risk factor modules was achieved by interview with health professionals and a systematic review of management options available in the local area. After identifying four consistent management options, each was then tailored for the four chosen risk factors. The management options included choices where the patient could take a more passive role and choices where the patient could take a more independent and active role in their health-care (self-help). Therefore, the choices within each module were designed to facilitate a successful outcome, or risk factor reduction, for a diverse range of personality types and for patients at different stages since their original heart event.

The information needs expressed by patients in phase two compared favourably with the suggestions given in published guidelines [17], for example, the need for specific and honest information, a concise design and the availability of treatment options. We addressed both process and content guidelines by involving patients and health professionals, reviewing clinical evidence and evaluating the leaflets. Therefore, this study provides an extension from published guidelines, documents the specific information needs of patients with CHD risk factors and highlights the importance of identifying patient needs using interview, as suggested by previous researchers [48].

When tested for quality, all four newly developed module information leaflets scored ratings greater than 'moderate' level on the DISCERN instrument. The leaflets were designed to provide basic information about the risk factor rather than comprehensive information about heart disease. All leaflets scored highly on items relating to treatment options, sources of additional information and support for shared decision-making. This finding confirms the pre-defined intention of the developed resources, particularly in terms of shared decision making.

When assessed for readability, the majority of leaflets scored in the 'fairly easy to read' Flesch category. The mean Flesch reading ease score for the leaflets was 75, indicating that 80% of the general population would be expected to understand it [35]. Many of the polysyllabic words (eg, blood cholesterol) appeared repetitively in the leaflets and hence the Flesch score probably underestimated reading ease. Importantly, studies comparing readability and knowledge, found that a leaflets reading ease was associated with a significant increase in knowledge [36,41].

The blood cholesterol leaflet scored the lowest readability, probably because the word 'cholesterol' was repeated nine times in the 100 word sample assessed. The word 'cholesterol' represents a four syllable word which would have adversely affected the Flesch score. Previous authors have criticised the use of readability formulas because they disregard patients' familiarity with certain words [39]. Importantly, when interviewed, no participants with high cholesterol identified the 'cholesterol' as a term they did not understand. A similar scenario existed for the physical activity leaflet. While the readability score is a useful objective method of assessing written education material, the scores do not consider patient motivation, visual attractiveness and conceptual background of the reader. Therefore, the score, as suggested by previous authors [49], was only used as one element for consideration when developing the written material.

When assessed for suitability, all leaflets scored in the category representing 'superior' suitability [57]. Each leaflet scored similarly because each followed the same basic style and layout and the SAM is weighted fairly heavily in terms of these aspects. The main deficiency in each of the information leaflets was the reading level score. To achieve the maximum score the reading level had to be of grade 5 or less. As described previously it is likely that the module information leaflets scored greater reading age then grade five due to the repetitive use of polysyllabic words such as "cholesterol" and "physical activity".

Compared to the newly developed module leaflets, the existing CR information leaflets scored poorly on all assessment tools. The CR leaflets were not dated, did not provide sources of additional information, presented an unbalanced view where only treatment was offered and they did not support shared decision making. Also, the CR leaflets were written in complex language and passive voice within minimal personalisation. These findings highlight the importance of involving patients throughout the process of development and demonstrate the superiority of the newly developed module information leaflets.

High scores for content and relevance suggest that people with relevant CHD risk factors found them useful and easy to comprehend. Overall, the cholesterol management leaflet tended to score slightly lower which may reflect the different nature of this module. Being a mandatory module, management choices were restricted. In contrast, the modules for BP, physical activity and smoking all had extensive information about treatment choices. The leaflets tended to score lowest on the section related to information about CHD but highest on the section about treatment choices. This finding reflects the aim of the leaflets which was to focus on flexibility and choice rather than to provide didactic information.

There are various strengths and weaknesses of the progressive approach used in this study. We have documented a clinically practical method for developing and testing patient information materials that involved patients and health professionals throughout. However, only a small number of participants were involved for each phase and most did not have severe CHD. As only four key risk factors were chosen for development into modules and corresponding leaflets, future research could investigate modules and leaflets for all modifiable CHD risk factors. Also, as the leaflets were only compared to the existing information leaflet from one cardiac rehabilitation program, comparison of the leaflets with patient information from a diverse range of cardiac rehabilitation programs would be beneficial.

## Conclusion

Using a three phase approach including qualitative and quantitative research methods, a series of risk factor modules and corresponding information leaflets have been developed and tested. Importantly, this study describes a readily applicable systematic process for designing and validating written patient information. This study also documents the unique perspective of persons with CHD risk factors. The leaflets are now considered suitable for use in further studies to test the effectiveness of a modular approach to secondary prevention of CHD and are expected to contribute to shared-decision making, patient empowerment and active patient participation.

## Competing interests

The author(s) declare that they have no competing interests.

## Authors' contributions

JR, EE, TB and BF were responsible for the design of the study. All authors read and approved the final manuscript.

## Pre-publication history

The pre-publication history for this paper can be accessed here:



## Figures and Tables

**Table 1:** Identified information needs of patients with heart disease

**Cholesterol**
Information about the recommended level
Information about own cholesterol
Information about how cholesterol causes heart disease
Information about the effect of diet and medication
Frequency of blood testing required
Duration for taking medication
Need to document own cholesterol and date of blood test
**Blood pressure (BP)**
Basic and easy to understand information about how BP effects the heart
Which medications are for BP
Need space available to record questions and communication with doctor
All claimed it would be motivating if the doctor suggested they decrease their BP
**Physical inactivity**
Request for flexibility and choice to suit lifestyle
Need for practical and simple information about how activity reduces heart disease
Information about safety and when to stop activity
Duration and frequency of activity
Suggestions such as having a chart (eg, on the fridge) would be motivating
**Smoking**
None said they need information about why they should stop
Practical suggestions and choice about cessation methods
Don't want to be told what to do
Recommendation from the doctor would be motivating
Information about nicotine patches
All stated that setting a quit date would be helpful
Knowing they were being monitored by a hospital staff member would be motivating

**Table 2:** Management options for BP, smoking cessation and physical activity

**Choice Options**	**BP Management**	**Smoking Cessation**	**Physical Activity**
Medical consultation	Follow doctors advice	Follow doctors advice	Follow doctors advice
Health professional-led intervention	Group session at local hospital	Local hospital dedicated program for smoking cessation	Six week supervised group exercise
intervention	Outpatient diet review		Community group walking program
Home program	Home medication, exercise program prescribed by doctor and allied health professional	Nil relevant	Written Home exercise program prescribed by allied health professional
Self-directed	Heartline	Quitline	Physical activity resources accessed at own discretion
	Private dietitian	Heartline	
	Reading, multi-media resources		

**Table 3:** Module and existing information leaflet scores for quality, readability and suitability

		Blood cholesterol (mean ± SD)	Blood pressure (mean ± SD)	Tobacco smoking (mean ± SD)	Physical activity (mean ± SD)	Combined new leaflets (mean ± SD)	Existing CR leaflets (mean ± SD)	*p*
Quality (DISCERN instrument)	Reliability†	3.7 ± 1.4	3.5 ± 1.3	3.5 ± 1.4	3.8 ± 1.2	3.6 ± 1.3	2.9 ± 1.9	0.002
	Choices†	3.9 ± 1.2	3.8 ± 1.2	4.2 ± 0.9	3.9 ± 1.2	3.9 ± 1.2	2.6 ± 2.0	0.000
	Overall rating†	4.0 ± 0.0	4.0 ± 0.0	4.0 ± 0.0	4.0 ± 0.0	4.0 ± 0.0	2.0 ± 0.0	0.000
	Final result §	61.5	59.0	62.0	61.5	60.5	43.0	-

Readability (Flesch)	Reading ease‡	68	75	82	77	78	32	0.015
	Description	standard	fairly easy	easy	fairly easy	fairly easy	difficult	-
	Human interest *	71	70	70	70	70	4	0.000
	Description	dramatic	dramatic	dramatic	dramatic	dramatic	scientific	-
	Population able to understand	75%	80%	86%	80%	79%	24%	0.000

Suitability (SAM) rating **		83%	81%	81%	81%	81%	23%	0.000

SAM = suitability assessment of materials, CR = cardiac rehabilitation.

† scored on a 5-point Likert scale from 1 (low quality) to 5 (high quality).

§scores out of a possible 80 where higher scores represent higher quality.

‡ scores out of a possible 100, higher scores represent greater ease of understanding.

* scores out of a possible 100, higher scores represent higher personal interest.

** sores out of a possible 100, higher scores represent greater suitability.

*p *= probability of test statistic for difference between the combined new leaflets and the existing CR leaflets

**Table 4:** Rating of content and relevance of module information leaflets by patients

	Blood Cholesterol (mean ± SD)	Blood Pressure (mean ± SD)	Tobacco Smoking (mean ± SD)	Physical Activity (mean ± SD)	Combined results (mean ± SD)
Age (years)	62 ± 21	55 ± 22	59 ± 21	57 ± 19	58 ± 21
% male	40	40	50	50	45

Overall rating †	4.2 ± 0.9	4.3 ± 0.7	4.3 ± 0.7	4.2 ± 0.6	4.3 ± 0.7
Heart disease †	3.6 ± 0.7	4.4 ± 0.7	4.5 ± 0.5	4.4 ± 0.5	4.2 ± 0.7
Treatment options †	4.5 ± 0.6	4.7 ± 0.4	4.7 ± 0.4	4.7 ± 0.4	4.6 ± 0.5
Style and readability †	4.3 ± 0.6	4.4 ± 0.7	4.6 ± 0.5	4.4 ± 0.5	4.4 ± 0.6

†scored on a 5-point Likert scale from 1 (low quality) to 5 (high quality)
